# The common truncation variant in pancreatic lipase related protein 2 (PNLIPRP2) is expressed poorly and does not alter risk for chronic pancreatitis

**DOI:** 10.1371/journal.pone.0206869

**Published:** 2018-11-08

**Authors:** Balázs Csaba Németh, Zsófia Gabriella Pesei, Eszter Hegyi, Ákos Szücs, Andrea Szentesi, Péter Hegyi, Mark E. Lowe, Miklós Sahin-Tóth

**Affiliations:** 1 Center for Exocrine Disorders, Department of Molecular and Cell Biology, Boston University Henry M. Goldman School of Dental Medicine, Boston, MA, United States of America; 2 First Department of Medicine, University of Szeged, Szeged, Hungary; 3 Institute for Translational Medicine, University of Pécs, Pécs, Hungary; 4 First Department of Surgery, Semmelweis University, Budapest, Hungary; 5 First Department of Medicine, University of Pécs, Pécs, Hungary; 6 Department of Pediatrics, Washington University School of Medicine, St Louis, MO, United States of America; University of Pittsburgh, UNITED STATES

## Abstract

A nonsense variant (p.W358X) of human pancreatic lipase related protein 2 (PNLIPRP2) is present in different ethnic populations with a high allele frequency. In cell culture experiments, the truncated protein mainly accumulates inside the cells and causes endoplasmic reticulum stress. Here, we tested the hypothesis that variant p.W358X might increase risk for chronic pancreatitis through acinar cell stress. We sequenced exon 11 of *PNLIPRP2* in a cohort of 256 subjects with chronic pancreatitis (152 alcoholic and 104 non-alcoholic) and 200 controls of Hungarian origin. We observed no significant difference in the distribution of the truncation variant between patients and controls. We analyzed mRNA expression in human pancreatic cDNA samples and found the variant allele markedly reduced. We conclude that the p.W358X truncation variant of *PNLIPRP2* is expressed poorly and has no significant effect on the risk of chronic pancreatitis.

## Introduction

Recurrent acute pancreatitis and chronic pancreatitis are inflammatory diseases of the pancreas with significant health and economic burdens [1, 2]. After an initial episode of acute pancreatitis, 10 to 30% of adults and children have additional episodes and, of those, a large fraction develop chronic pancreatitis (CP) [3, 4]. Progression of a single episode to chronic pancreatitis often associates with genetic risk factors in genes encoding digestive enzymes expressed in pancreatic acinar cells [5–7]. Since the discovery that a genetic variant in *PRSS1* (cationic trypsinogen) causes hereditary pancreatitis, most investigations to identify additional genetic risk factors focused on proteases and their inhibitors [8, 9].

More recent studies linked genetic variants in pancreatic lipases to increased risk for CP. The first report described variants in the gene encoding carboxyl ester lipase (*CEL*) that result in a form of autosomal dominant CP characterized by early-onset pancreatic insufficiency and diabetes [10]. A subsequent study found that a hybrid allele resulting from recombination of *CEL* and a neighboring pseudogene, *CELP*, increased risk for CP in northern Europeans [11]. Additionally, a report of two brothers who had a deficiency of pancreatic lipase (PNLIP) and evidence of CP showed they were homozygous for a missense mutation in *PNLIP* [12]. Follow-up studies indicated that the genetic variants of CEL and PNLIP likely cause disease through increased protein misfolding and maladaptive activation of unfolded protein response pathways [11, 13–15]. Importantly, these studies suggest that genetic variants in other pancreatic lipases, such as the pancreatic lipase related protein 2 (PNLIPRP2), might increase the risk for CP.

PNLIPRP2 is homologous with PNLIP and both belong to the same large lipase gene family [16–18]. Unlike PNLIP, which only digests triglycerides, PNLIPRP2 has lipase activity against triglycerides, phospholipids and galactolipids [16]. In newborn mice, PNLIPRP2 plays a critical role in fat digestion [19]. Its role in humans remains unclear. Intriguingly, a nonsense variant (p.W358X) in human *PNLIPRP2* is present in different ethnic populations at a high allele frequency of 0.3 to 0.5 [20]. When expressed in transfected HEK 293T cells, the truncated protein largely accumulated inside the cells as a detergent-insoluble aggregate and only a small amount was secreted into the medium [21]. The intracellular aggregates activated the unfolded protein response. The findings show that p.W358X PNLIPRP2 can alter cellular physiology through two mechanisms. First, the secretory defect results in a loss of function that might affect dietary fat digestion. Second, the intracellular aggregates of truncated PNLIPRP2 may result in a gain of function by placing pancreatic acinar cells at increased risk for injury through a maladaptive unfolded protein response. In combination with other stressors, the presence of PNLIPRP2 aggregates could activate cell death and inflammatory pathways leading to pancreatitis. A similar mechanism was reported for misfolding PRSS1 and carboxypeptidase A1 (CPA1) mutants, which appear to cause pancreatitis through endoplasmic reticulum stress [22]. Herein, we investigated whether the p.W358X *PNLIPRP2* allele is a genetic risk factor for CP in patients with alcohol-related and non-alcohol-related CP.

## Materials and methods

### Nomenclature

Nucleotide numbering follows coding DNA numbering with the first nucleotide of the ATG translation initiation codon designated as +1. Amino acids are numbered starting with the initiator methionine of the primary translation product of *PNLIPRP2*. The NCBI genomic reference sequence for *PNLIPRP2* (NC_000010.11, *Homo sapiens* chromosome 10, GRCh38.p12 primary assembly) and the NCBI coding DNA reference sequence (NM_005396.4) correspond to the minor truncation allele. In the present study, we used the major full-length *PNLIPRP2* allele as reference for the designation of all *PNLIPRP2* variants. In this manner, the nonsense p.W358X variant becomes the “effect” allele, which is the only biologically meaningful representation. Table 1 compares *PNLIPRP2* variant designations using the two different reference sequences and lists the dbSNP numbers for unambiguous identification.

**Table 1:** Designation of *PNLIPRP2* variants with respect to the NCBI reference sequence corresponding to the minor truncation allele and the full-length major allele used as the reference in this study.

		NCBI reference minor truncation allele		Reference used in this work major full-length allele	
*PNLIPRP2* region	dbSNP number	Nucleotide change	Amino acid change	Nucleotide change	Amino acid change
Intron 10		c.1070-379delG		c.1070-379delG	
Intron 10	rs4751994	c.1070-321C>T		c.1070-321T>C	
Exon 11	rs4751995	c.1074A>G	p.X358W	**c.1074G>A**	**p.W358X**
Exon 11	rs4751996	c.1084A>G	p.I362V	c.1084G>A	p.V362I
Exon 11	rs10885997	c.1161A>G	p.S387 =	c.1161G>A	p.S387 =
Intron 11	rs7910135	c.1181+55C>A		c.1181+55A>C	

The truncation variant is highlighted in bold type.

### Study subjects

This study used de-identified genomic DNA samples from the registry of the Hungarian Pancreatic Study Group (ethical approval number TUKEB 22254-1/2012/EKU; biobanking approval number IF702-19/2012). Subjects were recruited from 11 Hungarian centers between 2012 and 2018 and all gave informed consent according to the ethical guidelines of the Declaration of Helsinki. The current study was also approved by the Institutional Review Board at Boston University (“Analysis of susceptibility genes in patients with chronic pancreatitis”; IRB number H-35382). A total of 256 unrelated patients with CP, including 152 with alcoholic CP and 104 with non-alcoholic CP and 200 control subjects with no pancreatic disease were analyzed. The CP study cohort included patients with a history of recurrent acute pancreatitis and/or pathological imaging findings consistent with CP, such as pancreatic calcifications, duct dilatation or irregularities, with or without exocrine pancreatic insufficiency or diabetes. Patient characteristics are described in Table 2. Alcoholic CP was diagnosed in CP cases with alcohol consumption of more than 80 g/day (men) or 60 g/day (women) for at least two years. De-identified pancreatic cDNA and matching genomic DNA samples (n = 9) from cadaveric donors were obtained from the University of Szeged, Hungary.

**Table 2:** Study population.

	All CP *n = 256*		NACP n = 104		ACP n = 152		Controls *n = 200*	
	Male	Female	Male	Female	Male	Female	Male	Female
**number**	194	62	60	44	134	18	113	87
**mean age at recruitment**	56±10	56±14	57±12	57±16	55±10	53±9	52±12	52±13
**mean age of disease onset**	48±12	48±16	47±12	48±18	48±12	48±9	-	-

Age values indicate mean ± S.D. in years. CP, chronic pancreatitis, NACP, non-alcoholic chronic pancreatitis, ACP, alcoholic chronic pancreatitis.

### DNA sequencing

Primer sequences and amplicon sizes are listed in Table 3. PCR reactions were performed using 1.0 U HotStar Taq DNA polymerase (Qiagen, Valencia, CA), 0.2 mM dNTP, 2.0 μL 10x PCR buffer (Qiagen), 0.5 μM primers, and 10–50 ng genomic DNA or cDNA template in a total volume of 20 μL. Cycling conditions were as follows: 15-min initial heat activation at 95 ^o^C; 40 cycles of 30 s denaturation at 94 ^o^C, 30 s annealing at 60 ^o^C, and 60 s extension at 72 ^o^C; and final extension for 5 min at 72 ^o^C. Products were verified by 1.5% agarose gel electrophoresis. PCR amplicons (5 μL) were treated with 1 μL FastAP Thermosensitive Alkaline Phosphatase and 0.5 μL Exonuclease I (Thermo Fisher Scientific, Waltham, MA) for 15 min at 37 ^o^C and the reaction was stopped by heating the samples to 85 ^o^C for 15 min. Sanger sequencing was performed using the forward PCR primers as sequencing primer. Amplicons containing the heterozygous c.1070-379delG variant were also sequenced with the reverse primer.

**Table 3:** Oligonucleotide primers used for PCR amplification of exon 11 of *PNLIPRP2* from genomic DNA (e11 primers) and a portion of the *PNLIPRP2* coding sequence from pancreatic cDNA (RT primers).

Primer name	Sequence (5’>3’)	Amplicon	Annealing temperature
PNLIPRP2 e11 forward PNLIPRP2 e11 reverse	GTT CTG GAG GAT GGA AAT CTG CAA AAG GAG TTA GCA CAT GAC T	836 bp	60 ^o^C
PNLIPRP2 RT forward PNLIPRP2 RT reverse	CAT CTG GAT TTC TTT CCA AAT GG CGA GTG CAT TAA AGA TTT TAT TAC CG	732 bp	60 ^o^C

## Results

### A common truncation variant in *PNLIPRP2*

The common truncation variant c.1074G>A (p.W358X) in *PNLIPRP2* was first described in 2003 as W357X in European, African and Chinese populations with allele frequencies of 0.53, 0.55 and 0.33, respectively [20]. A 2010 study on the association of common gene variants and human dietary habits described the variant as W358X (rs4751995) with similar allele frequencies [23]. The discrepancy in numbering is because the original cloning study of *PNLIPRP2* missed one of the two consecutive Met codons at the start of the coding sequence [18]. Interestingly, the first Met is encoded by a separate upstream exon, which should be counted as exon 1 of the *PNLIPRP2* gene; placing the truncation variant in exon 11. The NCBI reference sequence for *PNLIPRP2* corresponds to the minor truncation allele. To describe the truncation variant in a biologically meaningful manner, in this study we used the major full-length *PNLIPRP2* allele as reference (Table 1).

### DNA sequence analysis of exon 11 of human *PNLIPRP2*

**Fig 1:**
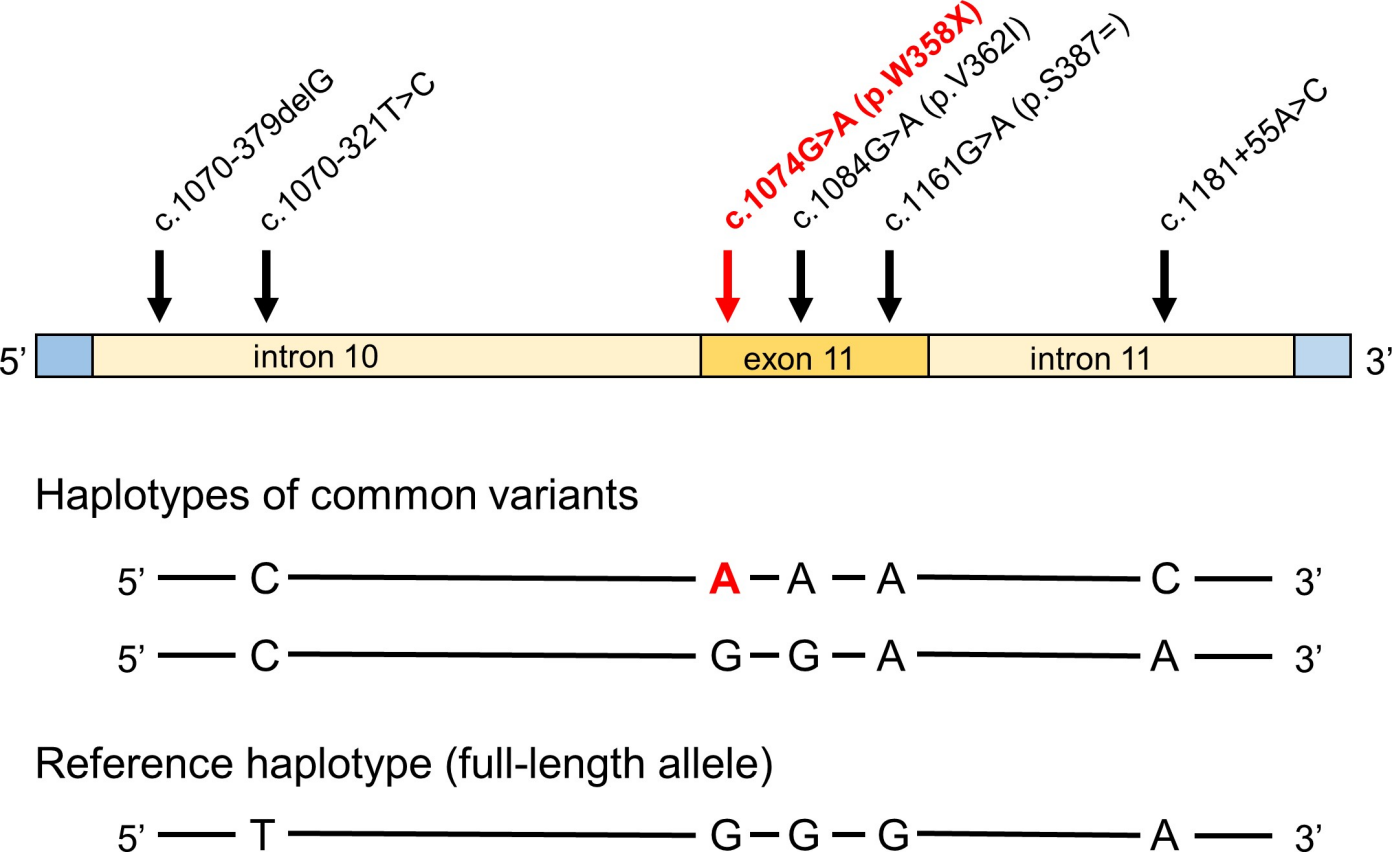
*PNLIPRP2* variants in exon 11 and the flanking intronic regions identified in the present study. The truncation variant is in bold type. The three haplotypes formed by the five commonly occurring variants are also shown.

We genotyped 152 subjects with alcoholic CP, 104 subjects with non-alcoholic CP and 200 control subjects, recruited from the registry of the Hungarian Pancreatic Study Group. We used direct DNA sequencing after PCR amplification of exon 11 and flanking intronic regions of *PNLIPRP2*. Within the amplified 793 nt sequence, we found 6 nucleotide variants, which included three intronic variants (c.1070-379delG, c.1070-321T>C and c.1181+55A>C), one synonymous variant (c.1161G>A, p.S387 = ), one missense variant (c.1084G>A, p.V362I) and the truncation variant c.1074G>A (p.W358X) (Fig 1). The commonly occurring variants c.1070-321T>C, p.W358X, p.V362I, p.S387 = and c.1181+55A>C were found in linkage disequilibrium as a conserved haplotype (CAAAC in Fig 1). Another common haplotype (CGGAA in Fig 1) was formed by variants c.1070-321T>C and p.S387 = .

When allele frequency was considered, distribution of the variants between patients and controls showed no significant difference (Table 4). Subgroup analysis for alcoholic and non-alcoholic CP patients versus controls revealed no association either (Tables 5 and 6). We also analyzed genotypes using dominant and recessive models but found no significant differences in genotype frequencies between all CP patients or the alcoholic and non-alcoholic cohorts versus controls (Tables 7, 8 and 9). Finally, comparison of the three haplotypes between patients and controls yielded no significant differences with the exception of the CGGAA haplotype (see Fig 1), which was overrepresented in the non-alcoholic CP cohort relative to controls (OR 1.6, *P* 0.04) (Tables 10, 11 and 12). We consider this a spurious association due to limited sample size and chance.

**Table 4:** Allele frequency of *PNLIPRP2* variants in patients with chronic pancreatitis (CP) and controls without pancreatic disease.

*PNLIPRP2*	Nucleotide change	Amino acid change	CP patient alleles	Control alleles	OR	*P* value	95% CI
Intron 10	c.1070-379delG		2/512 (0.4%)	1/400 (0.3%)	1.6	0.72	0.14–17.3
Intron 10	c.1070-321T>C		319/512 (62.3%)	240/400 (60%)	1.1	0.48	0.84–1.4
**Exon 11**	**c.1074G>A**	**p.W358X**	**245/512 (47.9%)**	**192/400 (48%)**	**0.99**	**0.97**	**0.77–1.3**
Exon 11	c.1084G>A	p.V362I	245/512 (47.9%)	192/400 (48%)	0.99	0.97	0.77–1.3
Exon 11	c.1161G>A	p.S387 =	321/512 (62.7%)	240/400 (60%)	1.1	0.4	0.86–1.5
Intron 11	c.1181+55A>C		246/512 (48%)	192/400 (48%)	1	0.99	0.77–1.3

The truncation variant is highlighted in bold type. OR, odds ratio; CI, confidence interval.

**Table 5:** Allele frequency of *PNLIPRP2* variants in patients with non-alcoholic chronic pancreatitis (NACP) and controls without pancreatic disease.

*PNLIPRP2*	Nucleotide change	Amino acid change	NACP patient alleles	Control alleles	OR	*P* value	95% CI
Intron 10	c.1070-379delG		1/208 (0.5%)	1/400 (0.3%)	1.9	0.64	0.12–31
Intron 10	c.1070-321T>C		131/208 (63%)	240/400 (60%)	1.1	0.48	0.8–1.6
**Exon 11**	**c.1074G>A**	**p.W358X**	**93/208 (44.7%)**	**192/400 (48%)**	**0.88**	**0.44**	**0.63–1.2**
Exon 11	c.1084G>A	p.V362I	93/208 (44.7%)	192/400 (48%)	0.88	0.44	0.63–1.2
Exon 11	c.1161G>A	p.S387 =	131/208 (63%)	240/400 (60%)	1.1	0.48	0.8–1.6
Intron 11	c.1181+55A>C		94/208 (45.2%)	192/400 (48%)	0.89	0.51	0.64–1.3

The truncation variant is highlighted in bold type. OR, odds ratio; CI, confidence interval.

**Table 6:** Allele frequency of *PNLIPRP2* variants in patients with alcoholic chronic pancreatitis (ACP) and controls without pancreatic disease.

*PNLIPRP2*	Nucleotide change	Amino acid change	ACP patient alleles	Control alleles	OR	*P* value	95% CI
Intron 10	c.1070-379delG		1/304 (0.3%)	1/400 (0.3%)	1.3	0.85	0.08–21.1
Intron 10	c.1070-321T>C		188/304 (61.8%)	240/400 (60%)	1.1	0.62	0.8–1.5
**Exon 11**	**c.1074G>A**	**p.W358X**	**152/304 (50%)**	**192/400 (48%)**	**1.1**	**0.6**	**0.8–1.5**
Exon 11	c.1084G>A	p.V362I	152/304 (50%)	192/400 (48%)	1.1	0.6	0.8–1.5
Exon 11	c.1161G>A	p.S387 =	190/304 (62.5%)	240/400 (60%)	1.1	0.5	0.82–1.5
Intron 11	c.1181+55A>C		152/304 (50%)	192/400 (48%)	1.1	0.6	0.8–1.5

The truncation variant is highlighted in bold type. OR, odds ratio; CI, confidence interval.

**Table 7:** Genotype distribution of *PNLIPRP2* variants in patients with chronic pancreatitis (CP) and in controls.

*PNLIPRP2*	Nucleotide change	Genotype	CP patients	Controls	OR	*P* value	95% CI
Intron 10	c.1070-379delG	GG delG deldel	254/256 (99.2%) 2/256 (0.8%) 0/256 (0%)	199/200 (99.5%) 1/200 (0.5%) 0/200 (0%)	*1*.*6* 0.78	*0*.*72* 0.9	*0*.*14–17*.*4* 0.02–39.6
Intron 10	c.1070-321T>C	TT TC CC	37/256 (14.5%) 119/256 (46.5%) 100/256 (39%)	27/200 (13.5%) 106/200 (53%) 67/200 (33.5%)	*0*.*92* 1.3	*0*.*77* 0.22	*0*.*54–1*.*6* 0.87–1.9
**Exon 11**	**c.1074G>A**	GG GA AA	68/256 (26.6%) 131/256 (51.2%) 57/256 (22.2%)	50/200 (25%) 108/200 (54%) 42/200 (21%)	*0*.*92* 1.1	*0*.*7* 0.75	*0*.*6–1*.*4* 0.69–1.7
Exon 11	c.1084G>A	GG GA AA	68/256 (26.6%) 131/256 (51.2%) 57/256 (22.2%)	50/200 (25%) 108/200 (54%) 42/200 (21%)	*0*.*92* 1.1	*0*.*7* 0.75	*0*.*6–1*.*4* 0.69–1.7
Exon 11	c.1161G>A	GG GA AA	37/256 (14.5%) 117/256 (45.7%) 102/256 (39.8%)	27/200 (13.5%) 106/200 (53%) 67/200 (33.5%)	*0*.*92* 1.3	*0*.*77* 0.16	*0*.*54–1*.*6* 0.89–1.9
Intron 11	c.1181+55A>C	AA AC CC	68/256 (26.6%) 130/256 (50.8%) 58/256 (22.6%)	50/200 (25%) 108/200 (54%) 42/200 (21%)	*0*.*92* 1.1	*0*.*7* 0.67	*0*.*6–1*.*4* 0.7–1.7

Data were analyzed assuming dominant (shown in italics) or recessive models of inheritance. The truncation variant is highlighted in bold type. OR, odds ratio; CI, confidence interval.

**Table 8:** Genotype distribution of *PNLIPRP2* variants in patients with non-alcoholic chronic pancreatitis (NACP) and in controls.

*PNLIPRP2*	Nucleotide change	Genotype	NACP patients	Controls	OR	*P* value	95% CI
Intron 10	c.1070-379delG	GG delG deldel	103/104 (99%) 1/104 (1%) 0/104 (0%)	199/200 (99.5%) 1/200 (0.5%) 0/200 (0%)	*1*.*9* 1.9	*0*.*64* 0.75	*0*.*12–31*.*2* 0.04–97.4
Intron 10	c.1070-321T>C	TT TC CC	12/104 (11.5%) 53/104 (51%) 39/104 (37.5%)	27/200 (13.5%) 106/200 (53%) 67/200 (33.5%)	*1*.*2* 1.2	*0*.*63* 0.49	*0*.*58–2*.*5* 0.73–2
**Exon 11**	**c.1074G>A**	GG GA AA	26/104 (25%) 63/104 (60.6%) 15/104 (14.4%)	50/200 (25%) 108/200 (54%) 42/200 (21%)	*1* 0.63	*1* 0.17	*0*.*58–1*.*7* 0.33–1.2
Exon 11	c.1084G>A	GG GA AA	26/104 (25%) 63/104 (60.6%) 15/104 (14.4%)	50/200 (25%) 108/200 (54%) 42/200 (21%)	*1* 0.63	*1* 0.17	*0*.*58–1*.*7* 0.33–1.2
Exon 11	c.1161G>A	GG GA AA	12/104 (11.5%) 53/104 (51%) 39/104 (37.5%)	27/200 (13.5%) 106/200 (53%) 67/200 (33.5%)	*1*.*2* 1.2	*0*.*63* 0.49	*0*.*58–2*.*5* 0.73–2
Intron 11	c.1181+55A>C	AA AC CC	26/104 (25%) 62/104 (59.6%) 16/104 (15.4%)	50/200 (25%) 108/200 (54%) 42/200 (21%)	*1* 0.68	*1* 0.24	*0*.*58–1*.*7* 0.36–1.3

Data were analyzed assuming dominant (shown in italics) or recessive models of inheritance. The truncation variant is highlighted in bold type. OR, odds ratio; CI, confidence interval.

**Table 9:** Genotype distribution of *PNLIPRP2* variants in patients with alcoholic chronic pancreatitis (ACP) and in controls.

*PNLIPRP2*	Nucleotide change	Genotype	ACP patients	Controls	OR	*P* value	95% CI
Intron 10	c.1070-379delG	GG delG deldel	151/152 (99.3%) 1/152 (0.7%) 0/152 (0%)	199/200 (99.5%) 1/200 (0.5%) 0/200 (0%)	*1*.*3* 1.3	*0*.*85* 0.89	*0*.*08–21*.*2* 0.03–66.6
Intron 10	c.1070-321T>C	TT TC CC	25/152 (16.5%) 66/152 (43.4%) 61/152 (40.1%)	27/200 (13.5%) 106/200 (53%) 67/200 (33.5%)	*0*.*79* 1.3	*0*.*44* 0.2	*0*.*44–1*.*4* 0.86–2.1
**Exon 11**	**c.1074G>A**	GG GA AA	42/152 (27.6%) 68/152 (44.8%) 42/152 (27.6%)	50/200 (25%) 108/200 (54%) 42/200 (21%)	*0*.*87* 1.4	*0*.*58* 0.15	*0*.*54–1*.*4* 0.88–2.4
Exon 11	c.1084G>A	GG GA AA	42/152 (27.6%) 68/152 (44.8%) 42/152 (27.6%)	50/200 (25%) 108/200 (54%) 42/200 (21%)	*0*.*87* 1.4	*0*.*58* 0.15	*0*.*54–1*.*4* 0.88–2.4
Exon 11	c.1161G>A	GG GA AA	25/152 (16.5%) 64/152 (42.1%) 63/152 (41.4%)	27/200 (13.5%) 106/200 (53%) 67/200 (33.5%)	*0*.*79* 1.4	*0*.*44* 0.13	*0*.*44–1*.*4* 0.9–2.2
Intron 11	c.1181+55A>C	AA AC CC	42/152 (27.6%) 68/152 (44.8%) 42/152 (27.6%)	50/200 (25%) 108/200 (54%) 42/200 (21%)	*0*.*87* 1.4	*0*.*58* 0.15	*0*.*54–1*.*4* 0.88–2.4

Data were analyzed assuming dominant (shown in italics) or recessive models of inheritance. The truncation variant is highlighted in bold type. OR, odds ratio; CI, confidence interval.

**Table 10:** Distribution of common *PNLIPRP2* haplotype alleles in patients with chronic pancreatitis (CP) and in controls.

Haplotype	All CP patients	Controls	OR	*P* value	95% CI
**CAAAC**	**244/512 (47.7%)**	**191/400 (47.8%)**	**1**	**0.98**	**0.77–1.3**
CGGAA	73/512 (14.3%)	48/400 (12.0%)	1.2	0.32	0.83–1.8
TGGGA	191/512 (37.3%)	160/400 (40.0%)	0.89	0.41	0.68–1.2

The truncation haplotype is highlighted in bold type. OR, odds ratio; CI, confidence interval. See Fig 1 for more details.

**Table 11:** Distribution of common *PNLIPRP2* haplotype alleles in patients with non-alcoholic chronic pancreatitis (NACP) and in controls.

Haplotype	NACP patients	Controls	OR	*P* value	95% CI
**CAAAC**	**92/208 (44.2%)**	**191/400 (47.8%)**	**0.87**	**0.41**	**0.62–1.2**
CGGAA	38/208 (18.3%)	48/400 (12.0%)	1.6	0.040*	1.0–2.6
TGGGA	77/208 (37.0%)	160/400 (40.0%)	0.88	0.48	0.62–1.3

The truncation haplotype is highlighted in bold type. OR, odds ratio; CI, confidence interval. See Fig 1 for more details. The asterisk indicates significant association.

**Table 12:** Distribution of common *PNLIPRP2* haplotype alleles in patients with alcoholic chronic pancreatitis (ACP) and in controls.

Haplotype	ACP patients	Controls	OR	*P* value	95% CI
**CAAAC**	**152/304 (50.0%)**	**191/400 (47.8%)**	**1.1**	**0.55**	**0.81–1.5**
CGGAA	35/304 (11.5%)	48/400 (12.0%)	0.95	0.84	0.60–1.5
TGGGA	114/304 (37.5%)	160/400 (40.0%)	0.90	0.5	0.66–1.2

The truncation haplotype is highlighted in bold type OR, odds ratio; CI, confidence interval. See Fig 1 for more details.

### Expression of the *PNLIPRP2* truncation allele

**Fig 2:**
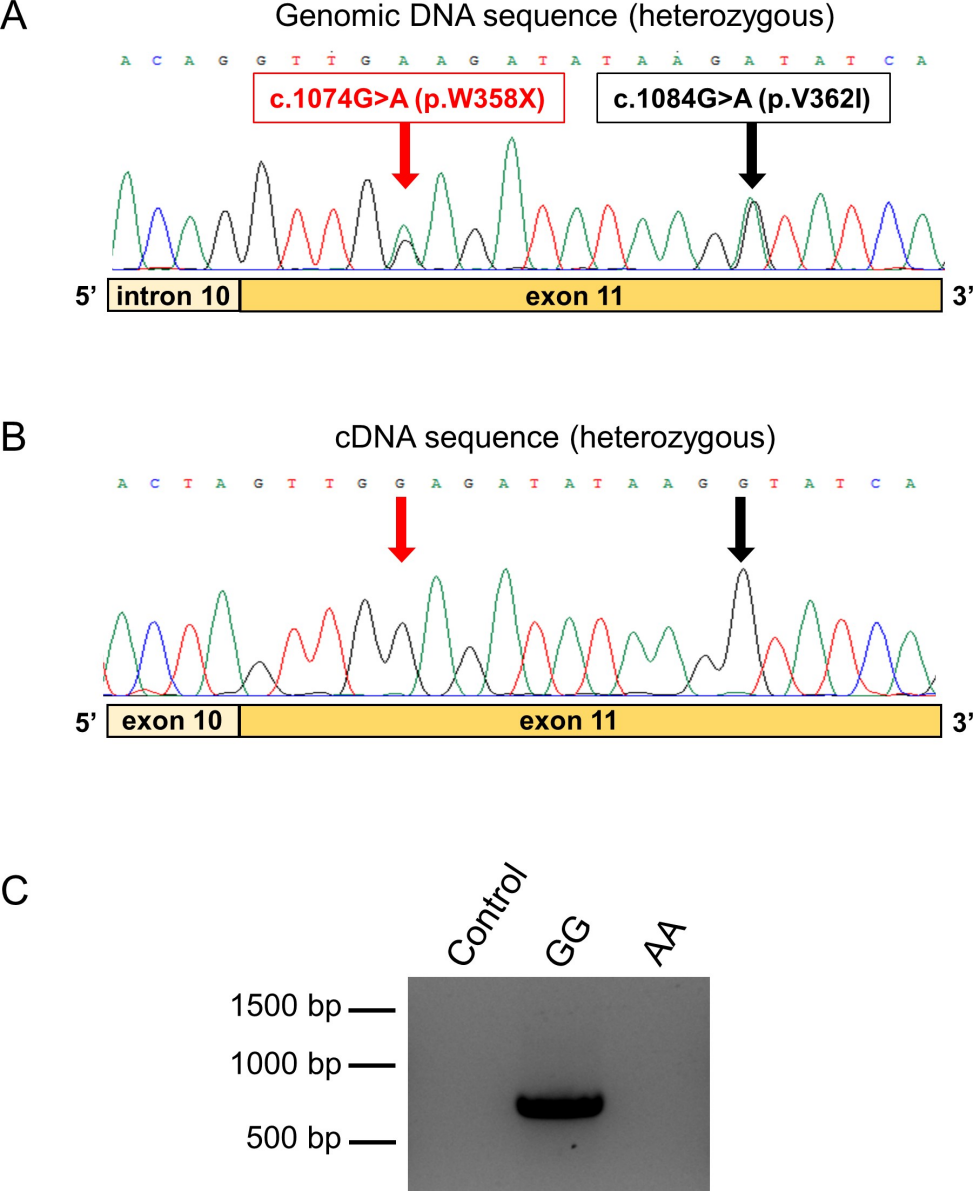
Expression of the *PNLIPRP2* p.W358X truncation variant. **A**, Electropherogram of the genomic DNA sequence of a heterozygous carrier showing the double signal at the position of variants p.W358X and p.V362I. **B**, Electropherogram of the pancreatic cDNA sequence of the same heterozygous subject. Note the absence of the signal corresponding to the minor truncation allele at the position of the variants. **C**, Agarose gel electrophoresis of PCR amplicons from pancreatic cDNA samples of subjects with homozygous A (minor truncation allele) and G (full-length allele) genotypes. Control reaction was performed with no added template.

To estimate the relative mRNA expression of the full-length and truncation alleles of *PNLIPRP2*, we used direct sequencing of pancreatic cDNA after PCR amplification of a 732 nt fragment of the coding DNA. We obtained nine de-identified cDNA samples with matching genomic DNA from cadaveric donors. Sequencing of the genomic DNA revealed five heterozygous samples and one sample homozygous for the truncation allele. The electropherograms of the heterozygous genomic sequences showed two signals at the position of variants p.W358X and p.V362I, with comparable peak heights (Fig 2A). Surprisingly, when heterozygous cDNA samples were sequenced, only one peak was visible at these positions, which corresponded to the major full-length allele, whereas no signal was apparent for the minor truncation allele (Fig 2B). PCR amplification of the pancreatic cDNA sample with the homozygous truncation allele confirmed the absence of detectable mRNA expression (Fig 2C). Taken together, our observations indicate that the truncation allele is not expressed at the mRNA level to a significant extent, in all likelihood due to nonsense-mediated mRNA decay.

**Fig 3:**
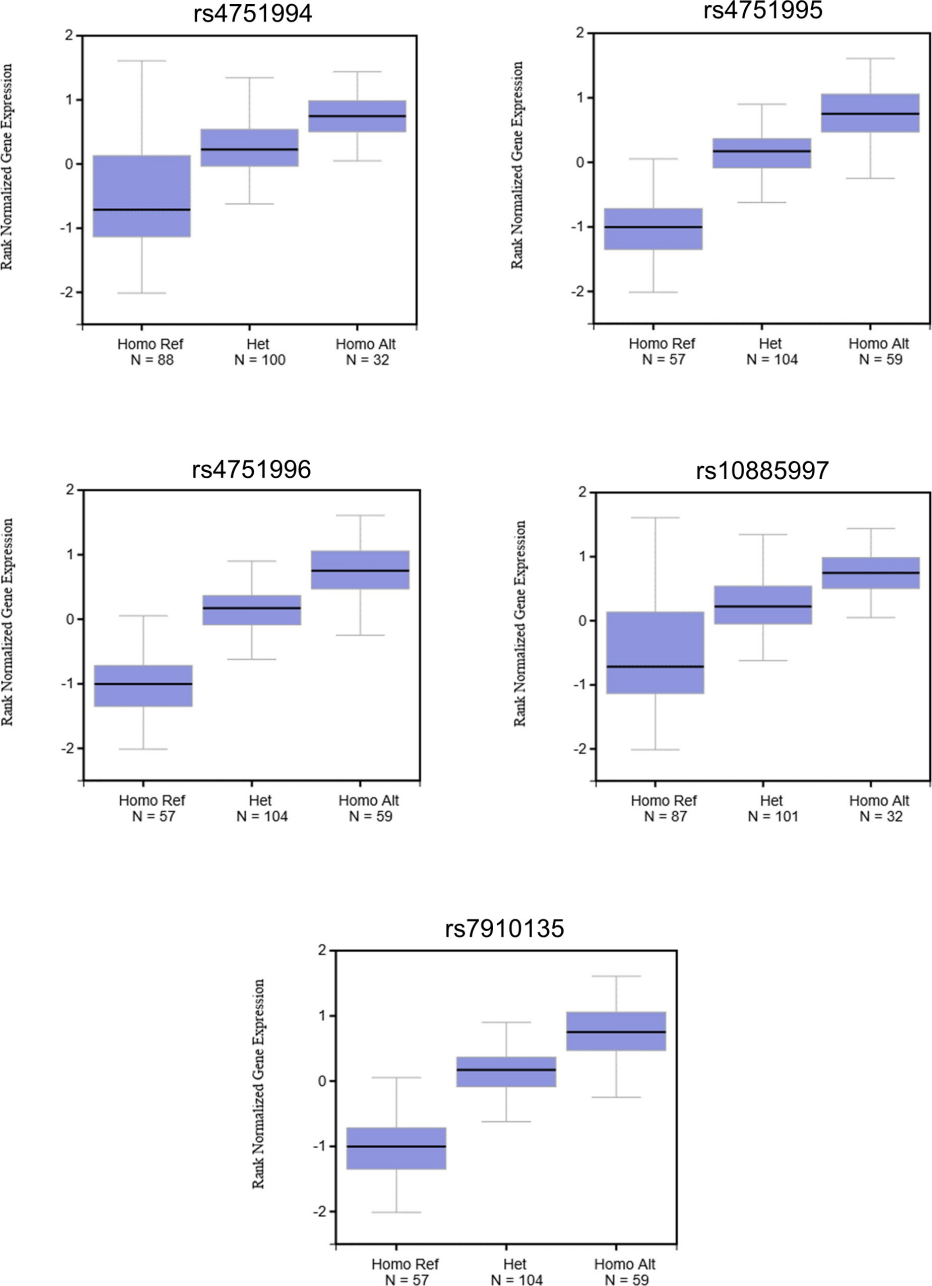
The effect of common *PNLIPRP2* variants on mRNA expression in the pancreas. Box plots were taken from the GTEx Portal (GTEx Analysis Release V7 - www.gtexportal.org). Note the diminished expression of the reference alleles (Ref), which correspond to the truncation haplotype in this database. See Table 1 for variant designation.

We also consulted the Genotype-Tissue Expression (GTEx) Portal (www.gtexportal.org/home) and found that all five common variants within the truncation haplotype were associated with diminished *PNLIPRP2* mRNA expression (Fig 3). The GTEx database is an open-access public resource to study tissue-specific gene expression and its relationship to genetic variation. The project analyzes global RNA expression within individual human tissues from deeply genotyped donors and correlates variations in gene expression with genetic alterations.

## Discussion

Physicians have increasingly recognized that CP is a complex disorder associated with multiple risk factors [24]. For many, particularly children, genetic variants in genes encoding pancreatic digestive enzymes contribute to the pathophysiology of CP [6]. In this study, we sought to determine if a common genetic variant in *PNLIPRP2* increased the risk for CP. The variant introduces a premature stop codon, p.W358X, resulting in a truncated protein and *in vitro* evidence suggests the expressed protein misfolds and activates the unfolded protein response [21]. We found no correlation of variant p.W358X with CP as a group or sub-grouped into alcoholic CP or non-alcoholic CP. This finding demonstrates that p.W358X is not a significant genetic risk factor for CP. We identified additional variants within exon 11 and the flanking intronic regions of *PNLIPRP2*, which formed conserved haplotypes. When these haplotypes were analyzed for disease association, we observed enrichment of the CGGAA haplotype (see Fig 1) in the non-alcoholic CP cohort. However, statistical significance was barely reached and we interpret this finding as fortuitous association due to the small sample size.

Because the presumed mechanism whereby variant p.W358X would contribute to CP is by activating maladaptive unfolded protein response and cell death pathways, we sought to determine if expression of the p.W358X allele was lower than expression of full length *PNLIPRP2*. If so, the levels of truncated protein may not be sufficient to activate the unfolded protein response. We accomplished this goal in two ways. First, we PCR amplified *PNLIPRP2* from pancreatic cDNA of heterozygous and homozygous p.W358X carriers and analyzed expression by DNA sequencing and agarose gel electrophoresis. Second, we interrogated the GTEx Portal database. Both methods confirmed that the amount of mRNA encoding p.W358X PNLIPRP2 is quite low compared to the mRNA amounts for full-length PNLIPRP2. The results suggest that the mRNA encoding the p.W358X variant undergoes nonsense-mediated decay [25]. In the previous study that characterized the cellular effects of the p.W358X variant the authors used artificial cDNA expression constructs, which lacked introns [21]. Consequently, the *PNLIPRP2* mRNA encoding the truncation variant did not suffer degradation and protein expression levels achieved were high enough to induce the unfolded protein response. The present data strongly argue that this cannot be the case when variant p.W358X is expressed from its native gene in the acinar cells.

Given the low levels of mRNA expression, it is unlikely that p.W358X PNLIPRP2 causes disease through gain-of-function as suggested by studies in transfected tissue culture cells [21]. In retrospect, it seems reasonable to have predicted that p.W358X PNLIPRP2 should not be a significant risk factor for CP or another disease since it is so prevalent. More likely, any effect of p.W358X PNLIPRP2 on human health should result from loss-of-function. Humans harbor many genetic variants predicted to cause loss-of-function [26]. Homozygosity for loss-of-function variants either results in a non-fatal phenotype or represent benign variations in redundant genes. A non-fatal loss-of-function phenotype was found in *Pnliprp2*-deficient mice [19]. Suckling *Pnliprp2*-deficient mice had fat malabsorption and poor growth but survived to adulthood and were fertile. It is not known if a similar effect occurs in human infants homozygous for p.W358X PNLIPRP2. In humans, p.W358X PNLIPRP2 may represent a loss-of-function tolerant genetic variant with the loss of its lipase activity compensated by other lipases [16]. Alternatively, p.W358X PNLIPRP2 may represent a protective or disease modifying allele [27]. That is, homozygosity for this allele may confer protection against disease or modify adaptations to diet [23]. Determination of the importance of the common p.W358X *PNLIPRP2* allele in human health will require additional investigations.
